# MIF Promotes Classical Activation and Conversion of Inflammatory Ly6C^high^ Monocytes into TipDCs during Murine Toxoplasmosis

**DOI:** 10.1155/2016/9101762

**Published:** 2016-02-29

**Authors:** Juan de Dios Ruiz-Rosado, Jonadab E. Olguín, Imelda Juárez-Avelar, Rafael Saavedra, Luis I. Terrazas, Frank H. Robledo-Avila, Alicia Vazquez-Mendoza, Jacquelina Fernández, Abhay R. Satoskar, Santiago Partida-Sánchez, Miriam Rodriguez-Sosa

**Affiliations:** ^1^Unidad de Biomedicina, Facultad de Estudios Superiores-Iztacala, UNAM, 54090 Tlalnepantla, MEX, Mexico; ^2^Laboratorio Nacional en Salud, Facultad de Estudios Superiores-Iztacala, UNAM, 54090 Tlanepantla, MEX, Mexico; ^3^Departamento de Inmunología, Instituto de Investigaciones Biomédicas, UNAM, CU, 04510 México City, DF, Mexico; ^4^Center for Microbial Pathogenesis, The Research Institute at Nationwide Children's Hospital, Columbus, OH 43205, USA; ^5^Carrera de Optometria, Facultad de Estudios Superiores-Iztacala, UNAM, 54090 Tlalnepantla, MEX, Mexico; ^6^Department of Pathology, The Ohio State University, Columbus, OH 43221, USA

## Abstract

Macrophage migration inhibitory factor (MIF) mediates immunity against* Toxoplasma gondii *infection by inducing inflammatory cytokines required to control the parasite replication. However, the role of this inflammatory mediator in the cell-mediated immune response against this infection is still poorly understood. Here, we used* T. gondii*-infected WT and* Mif* ^−/−^ mice to analyze the role of MIF in the maturation of CD11b^+^ and CD8*α* ^+^ dendritic cells (DCs). We found that MIF promotes maturation of CD11b^+^ but not CD8*α* ^+^ DCs, by inducing IL-12p70 production and CD86 expression. Infected* Mif* ^−/−^ mice showed significantly lower numbers of TNF and inducible nitric oxide synthase- (iNOS-) producing DCs (TipDCs) compared to infected WT mice. The adoptive transfer of Ly6C^high^ monocytes into infected WT or* Mif* ^−/−^ mice demonstrated that MIF participates in the differentiation of Ly6C^high^ monocytes into TipDCs. In addition, infected* Mif* ^−/−^ mice display a lower percentage of IFN-*γ*-producing natural killer (NK) cells compared to WT mice, which is associated with reducing numbers of TipDCs in* Mif* ^−/−^ mice. Furthermore, administration of recombinant MIF (rMIF) into* T. gondii*-infected* Mif* ^−/−^ mice restored the numbers of TipDCs and reversed the susceptible phenotype of* Mif* ^−/−^ mice. Collectively, these results demonstrate an important role for MIF inducing cell-mediated immunity to* T. gondii* infection.

## 1. Introduction

*Toxoplasma gondii* (*T. gondii*) is an obligate intracellular protozoan parasite that causes toxoplasmosis, a usually asymptomatic disease in most immune-competent individuals but potentially severe in immune-compromised hosts [1]. Successful control of this pathogen depends on the early production of the proinflammatory cytokines IL-12 and IFN-*γ*, required for the development of a protective cell-mediated immune response against the parasite [2–5].

Although neutrophils and macrophages produce IL-12 in response to* T. gondii* infection or its soluble antigens (STAg) [6–9],* in vivo* studies indicate that CD8*α* ^+^ and CD11b^+^ dendritic cell (DC) populations may be the most important sources of this proinflammatory cytokine during acute infection [10–12]. The strong IL-12-driven response against* T. gondii* results in the systemic production of IFN-*γ* by natural killer (NK) CD4^+^ and CD8^+^ T cells, which promotes the effector mechanisms to control both acute and chronic infections [2, 13–18].

MIF is a pleiotropic inflammatory cytokine that plays a role in the outcome of a variety of protozoan infectious diseases [19–23]. We have previously demonstrated that BALB/c* Mif* ^−/−^ mice were more susceptible to* T. gondii* infection, with the virulent RH strain and the avirulent ME49 strain [24]. The increased susceptibility in* Mif* ^−/−^ mice was associated with deficient serum levels of inflammatory cytokines (IL-12 and IFN-*γ*), as well as impaired IL-12 production and reduced expression of costimulatory molecules in CD11c^+^ cells from spleen or mesenteric lymph nodes [25]. These data indicated a deficient innate immune response on infected* Mif* ^−/−^ mice presumably due to a deficient DC maturation. However, as macrophages are able to express CD11c [26] and different DCs subsets share the expression of this marker, it is difficult to discern whether MIF has a role in the maturation of DC subsets involved in the resistance to* T. gondii*.

Therefore, the present study aimed to assess the role of MIF in the maturation of conventional CD8*α* ^+^ and CD11b^+^ DCs during the acute infection with avirulent* T. gondii* ME49 strain. We demonstrate that serum MIF increased rapidly during the acute infection with* T. gondii* and promoted maturation of CD11b^+^ but not CD8*α* ^+^ DCs. In addition, MIF promoted classical activation of inflammatory Ly6C^high^ monocytes and induced TipDCs. Adoptive transfer of bone marrow Ly6C^high^ monocytes suggested that systemic MIF levels are required for conversion of this monocyte subset into TipDCs.* Mif* ^−/−^ mice displayed reduced IFN-*γ* ^+^ natural killer (NK) cells compared to infected WT mice, suggesting that MIF promotes the recruitment of IFN-*γ*-producing NK cells. Finally, administration of rMIF to* T. gondii*-infected* Mif* ^−/−^ mice restored the numbers of TipDCs and inflammatory cytokines along with the resistance to the parasite. Our results uncover a new role for MIF in the development of cell-mediated immunity to* T. gondii* by favoring TipDC differentiation during acute toxoplasmosis.

## 2. Materials and Methods

### 2.1. Ethics Statement

All experiments in this study were performed strictly according to the guidelines for the Care and Use of Laboratory Animals adopted by the US National Institutes of Health and the Mexican Regulation of Animal Care (NOM-062-ZOO-1999, 2001). Protocols were also approved by the Institutional Animal Care and Use Committee (IACUC), The Research Institute at Nationwide Children's Hospital. All efforts were made to minimize animal suffering during the course of these studies.

### 2.2. Mice

Adult 6- to 8-week-old female mice (BALB/c background) were purchased from Harlan Laboratory (México or USA).* Mif* ^−/−^ mice were developed as previously described [27] and backcrossed for more than 10 generations to a BALB/c genetic background. All animals were maintained in a pathogen-free environment and established as breeding colonies in the Animal Facility at FES-Iztacala, Universidad Nacional Autónoma de México (UNAM), or in the Transgenic Mouse Facility at The Research Institute at Nationwide Children's Hospital animal facility.

### 2.3. Parasites and Experimental Infection

Cysts of the avirulent* T. gondii* ME49 strain were harvested from brains of C57BL/6 mice that had been inoculated* i.p.* with 40 cysts 2 months earlier. For experimental infections,* Mif* ^−/−^ or WT mice received via intraperitoneal (*i.p.*) 100 cysts or PBS as a control. Control inoculations with uninfected brains failed to elicit detectable inflammatory responses or significant increases in cytokine levels.

### 2.4. Analysis of the Acute Infections

To assess acute disease progression, mice were killed by CO_2_ and the peritoneal fluids were recovered under sterile condition using 3 mL of warm PBS and gentle massage at days 5 and 7 after toxoplasma infection of* Mif* ^−/−^ and WT mice. Tachyzoites were counted by trypan blue exclusion with a Neubauer hemocytometer under Olympus BX51 microscope (×100; Olympus American, Melville, NY) equipped with a digital video camera.

### 2.5. Cytokine Assays

Serum was obtained from infected or uninfected* Mif* ^−/−^ and WT mice during the acute phase of infection with* T. gondii* (1, 2, 3, 5, and 7 days after infection). The whole blood was centrifuged for 10 min at 2000 ×g at 4°C. MIF levels were determined by ELISA according to the manufacturer's guidelines (Neobiolab). Serum IFN-*γ*, IL-6, IL-12p70, IL-4, and IL-10 were measured using a Bio-Plex Pro Assay (BIO-RAD) or Cytometric Bead Array (CBA Th1/Th2, BD Biosciences) according to the manufacturer's protocols.

### 2.6. Flow Cytometry Analysis

Single-cell suspensions were obtained from peritoneal exudate cells (PECs) by peritoneal wash with 5 mL ice-cold PBS. PECs pellets were washed twice with complete RPMI 1640 media and filtered with 70 *μ*m Nylon Mesh (Fisher Scientific). Two milliliters of ACK lysing buffer (GIBCO, Life Technologies) was added to lyse red blood cells. Harvested cells were incubated in saturated doses of anti-mouse Fc receptor in 100 *μ*L of ice-cold FACS buffer (1% bovine serum albumin/0.01% NaN_3_ in PBS) for 15 min. After washing, 3 × 10^6^ cells were stained with various combinations of antibodies in ice-cold FACS buffer for 15 min and further collected on a LSR II cytofluorometer (Becton Dickinson, BD). Cells were gated according to size (SSC-A) and forward scatter (FSC-A). Blue-fluorescent reactive dye L23105 (Life Technologies) was used to discard dead cells. Absolute cell numbers were calculated with the total cell count multiplied successively by the percentages for the appropriate gates obtained through flow cytometry.

### 2.7. Intracellular Staining

Peritoneal cells free of red blood cells were washed with complete RPMI 1640 media and adjusted 3 × 10^6^ cells/mL. Two mL per well of the cell suspension was incubated in 24-well plate, stimulated with 5 ng/mL of Phorbol 12-myristate 13-acetate (PMA) (Sigma) plus 500 ng/mL of ionomycin (Sigma) and 10 *μ*g/mL of Brefeldin-A (Biolegend) at 37°C, 5% CO_2_ for 4 hours. Cells were recovered and placed into FACS tubes containing 1 mL of ice-cold FACS buffer and centrifuged at 1500 rpm for 5 min. After surface staining, cells were washed and fixed using 2% paraformaldehyde in PBS for 10 min, RT, darkness. Cells were washed with intracellular staining buffer (PBS, 1% FCS, and 0.025% saponin) and resuspended with 100 *μ*L of the same buffer containing the indicated mAbs for cytokines for 10 mins, RT, darkness. Cells were washed, resuspended in 500 *μ*L of buffer FACS, and analyzed in FACSAria Fusion cytometer (Becton Dickinson, BD).

### 2.8. Monoclonal Antibodies (mAbs) Used

The following mAbs were used: anti-CD11c Alexa Fluor 700 (N418) or anti-DEC-205 PE-cy7 (205yekta), anti-MHCII eFluor 450 (M5/114.15.2), anti-CD40 PE (1C10), anti-CD86 FITC (CT-CD8b), anti-CD86 Brilliant violet 605 (GL1), anti-CD11b Alexa Fluor 647 (M1/70), anti-Ly6C Alexa Fluor 488 (HK1.4), anti-F480 Alexa Fluor 488 (BM8), anti-Ly6C eFluor 450 (HK1.4), and anti-iNOS PE (CXNFT) from eBioscience; anti-CD8*α* Alexa Fluor 700 or PerCP (53-6.7), anti-CD11b Alexa Fluor 700 (M1/70), anti-F4/80 Brilliant violet 605 (BM8), anti-Ly6G PerCP (1A8), anti-CXCR4 Brilliant violet 421 (1276F12), anti-CXCR2 PE/Cy7 (5E8/CXCR2), anti-TNF-*α* Brilliant violet 650 (MP6-XT22), anti-CD3 FITC (145-2C11), anti-CD49 APC (DX5), and anti-IFN-*γ* PECy7 (XMG1.2) from Biolegend; and anti-MHC-II PE (AF6-120.1), anti-CD8*α* PerCP (53-6), anti-CD11c APC (N418), anti-CD74 FITC (In-1), anti-IL-12p40/70 V450 (C15.6), and anti-IL-12p40/70 APC (C15.6) from BD Bioscience.

### 2.9. Monocyte Isolation and Adoptive Transfer

Monocytes were isolated as previously described [28]. Briefly, bone marrow cell suspensions were isolated by flushing femurs and tibias from 6- to 8-week-old* Mif* ^−/−^ or WT BALB/c mice with complete RPMI 1640 supplemented with 10% fetal calf serum (FCS), 100 units of penicillin/streptomycin, 2 mM glutamine, 25 mM HEPES buffer, and 1% nonessential amino acids (all from GIBCO, BRL Grand Island, NY, USA). Bone marrow monocyte cells (BM-MNCs) were isolated using a density-gradient centrifugation method [29]. BM-MNCs were incubated with a mixture of antibody microbeads according to the manufacturer's protocol (Miltenyi Biotec, Auburn, CA). The cells were then run through LD-negative selection column. The negative fraction was collected (putative monocytes) and stained for cell sorting with a BD Influx Sorter. Isolated populations of Ly6C^high^ monocytes were obtained and stained with the cell trace proliferation dye eFluor 450 (Cat. number 65-0842-85; eBioscience) and transferred (2 × 10^6^ cells) intravenously (*i.v.*) to* T. gondii*-infected or uninfected* Mif* ^−/−^ or WT BALB/c mice.

### 2.10. Recombinant MIF Administration into* T. gondii*-Infected* Mif* ^−/−^ Mice

A group of 13* Mif* ^−/−^ and 13 WT mice were infected via* i.p.* with 100 cysts of* T. gondii*. Mice received (via* i.p.*) 0.5 *μ*g per mouse of rMIF (Biolegend #599504) daily during the first 5 days after* T. gondii* infection. Three mice of this group were selected randomly and sacrificed on days 3 and 5 after infection to analyze the presence of TipDcs and production of serum IFN-*γ* (as previously described). Uninfected mice that did not receive rMIF were used as a control. The rest of the group, 7* Mif* ^−/−^ and 7 WT mice, were monitored until day 25 to register the mortality and number of cysts per brain. Brain tissues were removed aseptically and homogenized in 2 mL of PBS. The total number of cysts was determined by counting cysts under Olympus BX51 microscope (×100; Olympus American, Melville, NY) equipped with a digital video camera (40x) in a 10 *μ*L aliquot and multiplied by 200.

### 2.11. Statistics

Statistical comparisons were performed by either Student's* t*-test or one-way ANOVA followed by Tukey's multiple comparisons for data that had a normal distribution. Graphed data are mean ± SD or SE. *P* values less than 0.05 were considered significant. All data were analyzed using the GraphPad prism 6 software.

## 3. Results

### 3.1. Experimental Acute Toxoplasmosis Increases Serum MIF

**Figure 1:**
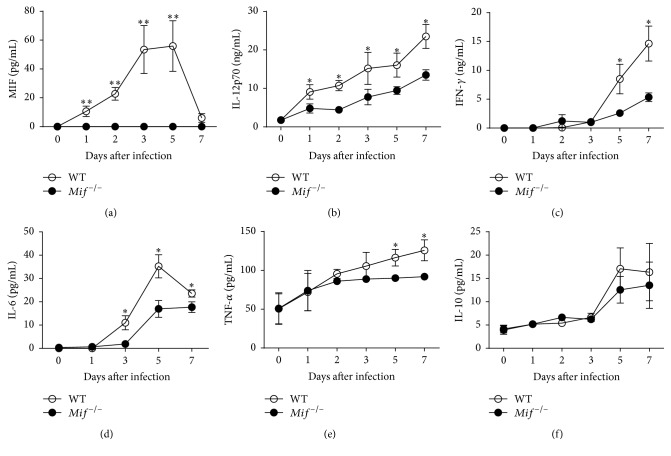
Serum MIF increases during acute* T. gondii* infection and regulates the production of proinflammatory cytokines. WT (○) and* Mif* ^−/−^ (●) mice were bled at various time points after infection with 100 cysts from ME49 strain. The serum was analyzed for cytokine concentrations by a Bio-Plex Pro Assay, Cytometric Bead Array (CBA), or MIF ELISA Kit. Data represent serum concentrations of MIF (a), IL-12p40 (b), IFN-*γ* (c), IL-6 (d), TNF-*α* (e), and IL-10 (f) through the course of infection from 2-3 independent experiments (*n* = 3–5 at each time point). Data are presented as the mean ± SE. ^*∗*^ *P* < 0.05.

Although MIF plays a critical role in the resistance to* T. gondii* infection [24], it is not known whether this protective role starts during the acute phase of infection with the parasite. Therefore, we first evaluated the systemic levels of inflammatory cytokines from acutely infected WT and* Mif* ^−/−^ mice with the avirulent ME49 strain from* T. gondii*. As shown in Figure 1(a), serum MIF from infected WT mice increased as early as 1 day after infection, peaked at 3 and 5 days, and decreased at day 7. In contrast,* Mif* ^−/−^ mice lacked serum MIF throughout the acute infection. The absence of MIF led to deficient levels of IL-12p70, IFN-*γ*, IL-6, and TNF-*α* in sera (Figures 1(b)–1(e)), but the anti-inflammatory cytokine IL-10 was similar between groups (Figure 1(f)). These data suggest that the early upregulation of MIF favors the prompt inflammatory immune response to acute toxoplasmosis.

### 3.2. MIF Induces Maturation of Conventional CD11b^+^ but Not CD8*α* ^+^ DCs during Acute* T. gondii* Infection

Under conditions of infection, DCs mature by exposure to microbial agents or inflammatory mediators and increase their antigen processing capacity as well as their expression of costimulatory molecules and cytokine production [30]. Recent studies in our laboratory suggest a role for MIF in inducing DC maturation from spleen or mesenteric lymph nodes during experimental toxoplasmosis [25]. Because DCs are essential for resistance to acute toxoplasmosis [10–12, 31], we evaluated the role of MIF in the maturation of conventional CD8*α* ^+^ and CD11b^+^ DC subsets in peritoneal exudate cells as the early primary response in the* i.p. T. gondii* infection.

**Figure 2:**
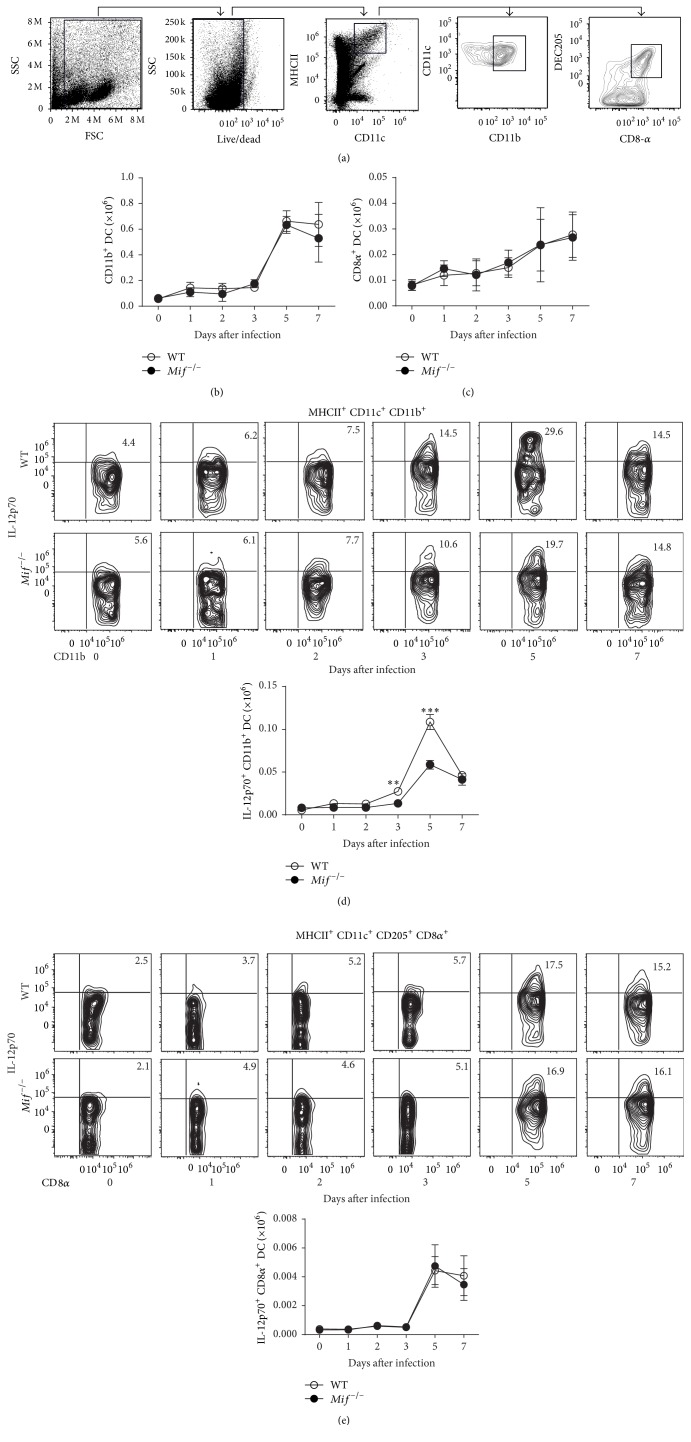
MIF induces IL-12 production on CD11b^+^ but not CD8*α* ^+^DCs during the acute infection with* T. gondii*. WT (○) and* Mif* ^−/−^ (●) mice were* i.p.* infected with 100 cysts of* T. gondii* ME49 strain, sacrificed on days 0, 1, 2, 3, 5, and 7 after infection, and analyzed for intracellular IL-12p70 in CD11b^+^ and CD8-*α* ^+^ DCs by flow cytometry. (a) Gating strategy of MHCII^+^ CD11c^+^ CD11b^+^ and MHCII^+^ CD11c^+^ CD205^+^ CD8*α* ^+^ DCs. (b) Kinetics of the absolute numbers of CD11b^+^ and (c) CD8*α* ^+^ DCs. (d) Representative FACs (upper panel) and analysis of absolute numbers (lower panel) of IL-12p70^+^CD11b^+^DCs. (e) Representative FACs (upper panel) and analysis of absolute numbers (lower panel) of IL-12p70^+^CD8*α* ^+^DCs. Data are representative of 2 independent experiments (*n* = 3 animals at each time point) and are expressed as mean ± SE. ^*∗*^ *P* < 0.05; ^*∗∗*^ *P* < 0.01.

In Figure 2(a), we show the gating strategy used for identification of CD11b^+^ (CD11c^+^ MHCII^+^ CD11b^+^) and CD8*α* ^+^ DCs (CD11c^+^ MHCII^+^ DEC205^+^ CD8*α* ^+^) subsets. The absolute numbers of both CD11b^+^ (Figure 2(b)) and CD8*α* ^+^ (Figure 2(c)) DCs increased at the site of infection (peritoneal cavity) until reaching a peak by day 5 after infection. Comparable numbers of these cell populations were observed between WT and* Mif* ^−/−^ mice. Nevertheless,* Mif* ^−/−^ mice displayed fewer IL-12p70^+^ CD11b^+^ DCs (Figure 2(d)), compared to WT mice at 3 (*P* = 0.0028) and 5 (*P* = 0.0006) days after infection. Similar numbers of IL-12p70^+^ CD8*α* ^+^ DCs were observed between* Mif* ^−/−^ and WT infected mice (Figure 2(e)).

**Figure 3:**
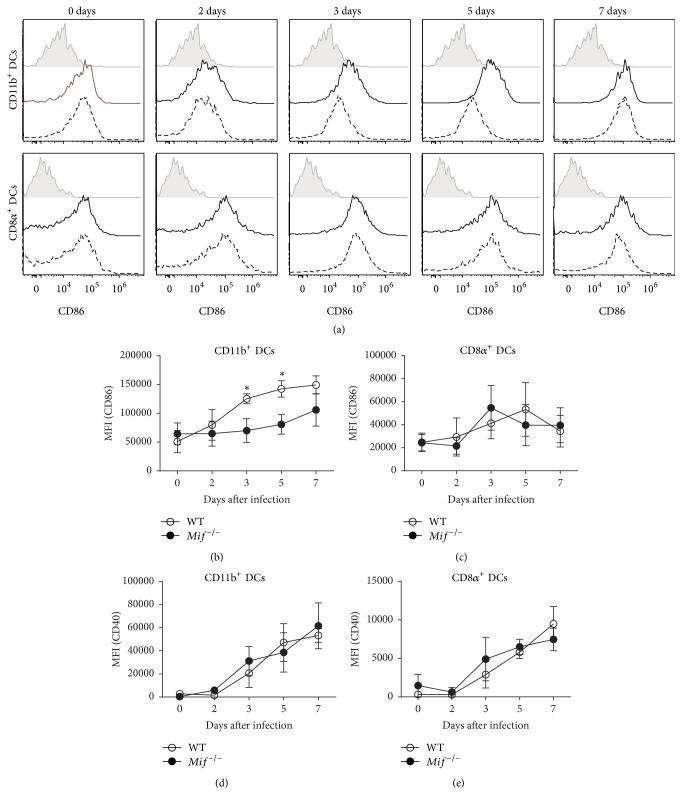
MIF induces CD86 expression on conventional CD11b^+^DCs but not CD8*α* ^+^DCs. Analysis of CD86 and CD40 expression on MHCII^+^ CD11c^+^ CD11b^+^ and MHCII^+^ CD11c^+^ CD205^+^ CD8*α* ^+^DCs from infected WT mice (solid line),* Mif* ^−/−^ mice (dashed line), and isotype (gray) at 0, 3, and 5 days after* T. gondii* infection. (a) Representative histograms of CD86 mean fluorescence intensity (MFI) in MHCII^+^ CD11c^+^ CD11b^+^ DCs and MHCII^+^ CD11c^+^ CD205^+^ CD8*α* ^+^DCs. (b) MFI kinetics of CD86 in CD11b^+^ DCs. (c) MFI kinetics of CD86 in CD8*α* ^+^ DCs. (d) MFI kinetics of CD40 in CD11b^+^ DCs. (e) MFI kinetics of CD40 in CD8*α* ^+^DCs from WT (○) and* Mif* ^−/−^ (●) (*n* = 3 animals at each time point, representative of 2 independent experiments). Data are presented as the mean ± SE. ^*∗*^ *P* < 0.05.

In addition,* Mif* ^−/−^ CD11b^+^ DCs displayed reduced CD86 expression (Figures 3(a) and 3(b)) at 3 (*P* = 0.0498) and 5 (*P* = 0.0194) days after infection, but no differences were observed in their expression of CD40 (Figure 3(d)). On the other hand, the expression of costimulatory molecules in CD8*α* ^+^ DCs was unaltered in the absence of MIF (Figures 3(a), 3(c), and 3(e)). Similar results were observed on DCs from the spleen (Figure S-1 in Supplementary Material available online at http://dx.doi.org/10.1155/2016/9101762). These findings suggest a role for MIF in selectively inducing maturation of CD11b^+^ DCs, a population that has proven to be crucial in the control of parasite replication and induction of the adaptive immune response against* T. gondii* [10].

### 3.3. MIF Induces TipDCs during Acute Toxoplasmosis

Previous studies have demonstrated that both iNOS and TNF-*α* are essential factors in the control of* T. gondii* infection. iNOS-deficient mice succumb during the chronic stage of infection with* T. gondii*, due to uncontrolled parasite replication, while TNF-*α*R-deficient mice are susceptible to both acute and chronic toxoplasmic encephalitis [4, 32–35].

**Figure 4:**
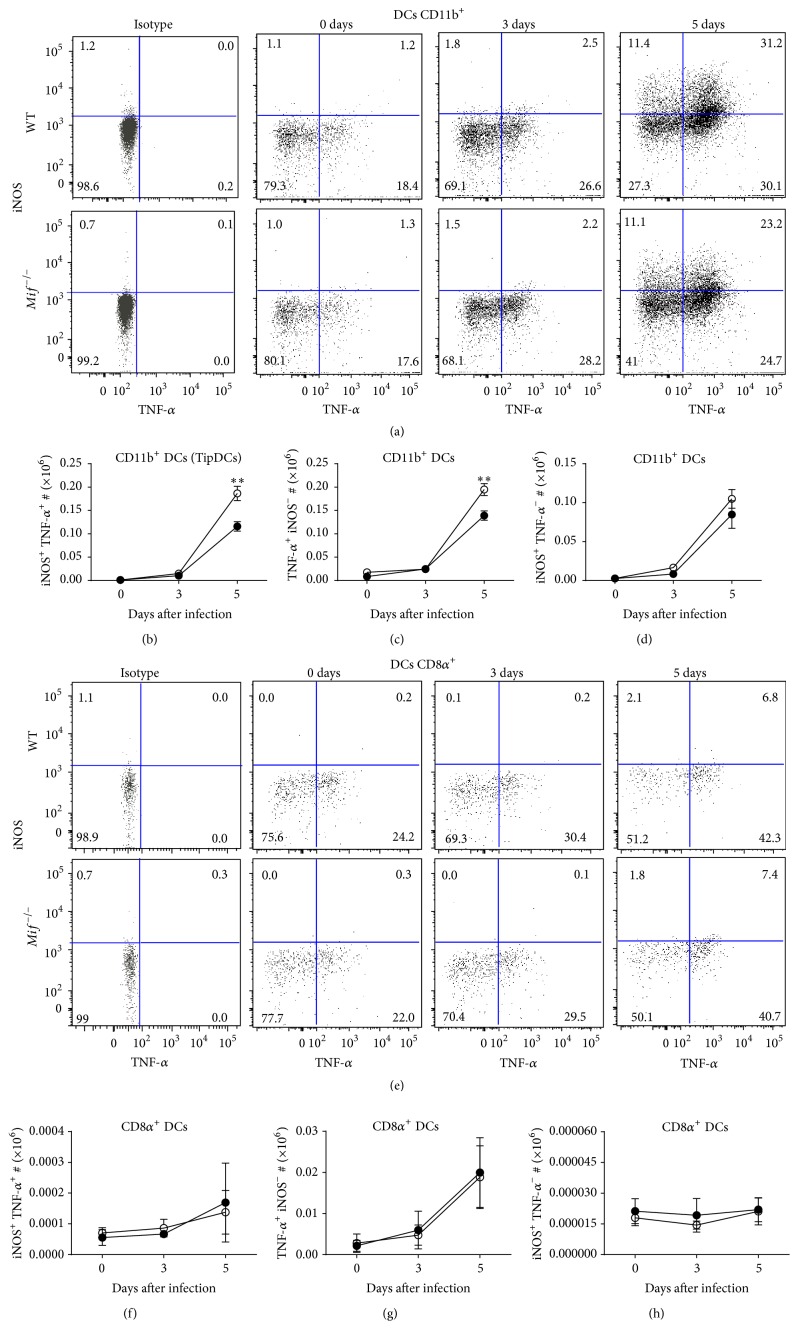
MIF induces TipDCS during acute* T. gondii* infection. CD11b^+^ and CD8*α* ^+^ DCs from WT (○) and* Mif* ^−/−^ (●) were analyzed for intracellular iNOS and TNF-*α* production at 0, 3, and 5 days after infection. (a) Representative dot plots from iNOS/TNF-*α*-producing CD11b^+^ (TipDCs). (b) Absolute numbers of iNOS^+^ TNF-*α* ^+^, (c) TNF-*α* ^+^ iNOS^−^, and (d) iNOS^+^ TNF-*α* ^−^ cells from CD11b^+^DCs. (e) iNOS/TNF-*α*-producing CD8*α* ^+^DCs. (f) Absolute numbers of iNOS^+^ TNF-*α* ^+^, (g) TNF-*α* ^+^iNOS^−^, and (h) iNOS^+^ TNF-*α* ^−^ cells from CD8*α* ^+^DCs during the acute phase of infection with* T. gondii* (*n* = 3 animals at each time point, representative of 2 independent experiments). Data are presented as the mean ± SE. ^*∗*^ *P* < 0.05; ^*∗∗*^ *P* < 0.01.

Monocyte-derived TNF-*α*/iNOS-producing CD11b^+^ DCs (TipDCs) can be induced during parasitic or microbial infections, and they exert potent antimicrobial functions required to control the infection [36–39]. However, the role of MIF in inducing TipDCs during acute* T. gondii* infection has not been addressed. Therefore, using the same strategy for identification of CD11b^+^ (CD11c^+^ MHCII^+^ CD11b^+^) and CD8*α* ^+^ (CD11c^+^ MHCII^+^ DEC205^+^ CD8*α* ^+^) DCs described above, we determined TNF-*α* and iNOS production on CD11b^+^ and CD8*α* ^+^ DCs from infected WT and* Mif* ^−/−^ mice. Our data revealed a population of TipDCs that gradually increased in both WT and* Mif* ^−/−^ mice during the acute phase of infection (Figure 4(a)). However, infected* Mif* ^−/−^ mice displayed reduced numbers of TipDCs (*P* = 0.0049; Figures 4(a) and 4(b)) and TNF-*α* ^+^ iNOS^−^ CD11b^+^ (*P* = 0.0090, Figures 4(a) and 4(c)) DCs at 5 days after infection. No differences were observed in iNOS^+^ TNF-*α* ^−^ CD11b^+^ DCs (Figures 4(a) and 4(d)).

CD8*α* ^+^ DCs showed a gradual increase in TNF-*α* (Figures 4(e)–4(h)), but not iNOS (Figures 4(f) and 4(h)), production during acute* T. gondii* infection. Comparable numbers of TNF-*α*-producing CD8*α* ^+^ DCs were observed between WT and* Mif* ^−/−^ mice (Figure 4(g)). These findings demonstrate a novel role for MIF in inducing TipDCs during acute toxoplasmosis and the ability of CD11b^+^ but not CD8*α* ^+^ DCs to mature into TipDCs.

### 3.4. Conventional CD11b^+^ DCs Express Greater Levels of MIF Receptors than CD8*α* ^+^ DCs

The chemokine-like inflammatory mediator MIF is a ligand for the single-pass type II membrane protein CD74 [40] and a noncognate ligand for the chemokine receptors CXCR2, CXCR4 [40], and CXCR7 [41]. Because the role of MIF in promoting DC maturation was only observed on CD11b^+^ DCs, we determined whether this phenomenon was associated with a differential expression of MIF receptors on CD11b^+^ and CD8*α* ^+^ DCs from infected WT mice.

**Figure 5:**
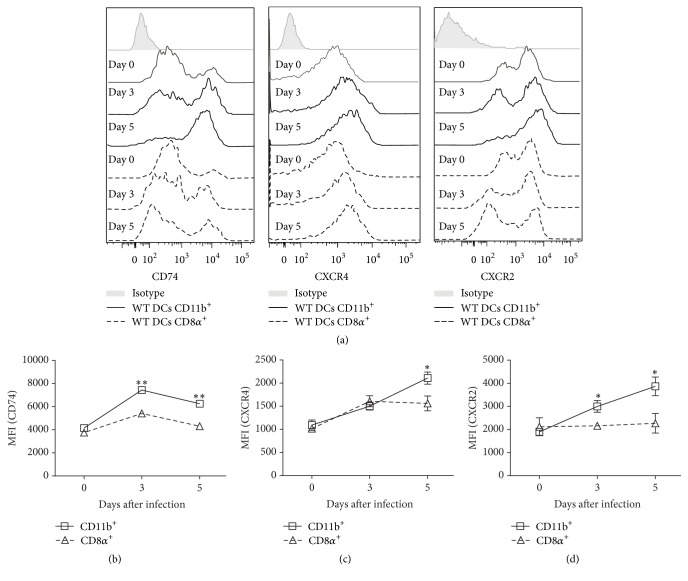
MIF receptors CXCR2, CXCR4, and CD74 are highly expressed on conventional CD11b^+^DCs but not CD8*α* ^+^DCs. Analysis of CXCR2, CXCR4, and CD74 expression on DCs from* T. gondii*-infected WT mice. (a) Representative histograms of CXCR2, CXCR4, and CD74 MFI in CD11b^+^(solid line) and CD8*α* ^+^(dashed line) DCs at 0, 3, and 5 days after infection. Kinetics of (b) CD74, (c) CXCR4, and (d) CXCR2 MFI in WT CD11b^+^(□) and WT CD8*α* ^+^(∆) DCs during acute* T. gondii* infection (*n* = 3 animals at each time point, representative of 2 independent experiments). Data are presented as the mean ± SE. ^*∗*^ *P* < 0.05; ^*∗∗*^ *P* < 0.01.

In basal conditions, WT CD11b^+^ and CD8*α* ^+^ DCs show similar expression of CD74, CXCR4, and CXCR2 (Figures 5(a), 5(b), and 5(c); day 0). However, during the course of* T. gondii* infection, WT CD11b^+^ DCs express more intensively the receptors CD74 (3 days, *P* = 0.0013; 5 days, *P* = 0.0014; Figure 5(b)), CXCR4 (5 days, *P* = 0.0014; Figure 5(c)), and CXCR2 (3 days, *P* = 0.0241; 5 days, *P* = 0.0341; Figure 5(d)), compared to WT CD8*α* ^+^ DCs (Figures 5(a)–5(d)). These results suggest that the selective role of MIF in the maturation of CD11b^+^ DCs may be related to the high expression of MIF receptors on this DC subset.

### 3.5. MIF Promotes Activation and Differentiation of Inflammatory Ly6C^high^ Monocytes during Acute* T. gondii* Infection

Two major subsets of circulating monocytes have been characterized in mice based on their phenotypical and functional characteristics. One subset expresses a high level of Ly6C^high^ and is referred to as inflammatory monocytes due to its early response and selective migration into inflamed tissues [42, 43]. The second subset, termed “resident” or “patrolling” monocytes, expresses a low level of Ly6C^low^ and is primarily distributed in blood vessels and tissues under the steady state, where it provides surveillance functions [42–45]. Because inflammatory Ly6C^high^ monocytes are able to differentiate into TipDCs [36, 39] and newly recruited Ly6C^+^ monocytes are required for resistance to oral or* i.p.* infection with* T. gondii* [46–50], we analyzed the activation of Ly6C^high^ and Ly6C^low^ monocytes in acutely infected* Mif* ^−/−^ and WT mice.

**Figure 6:**
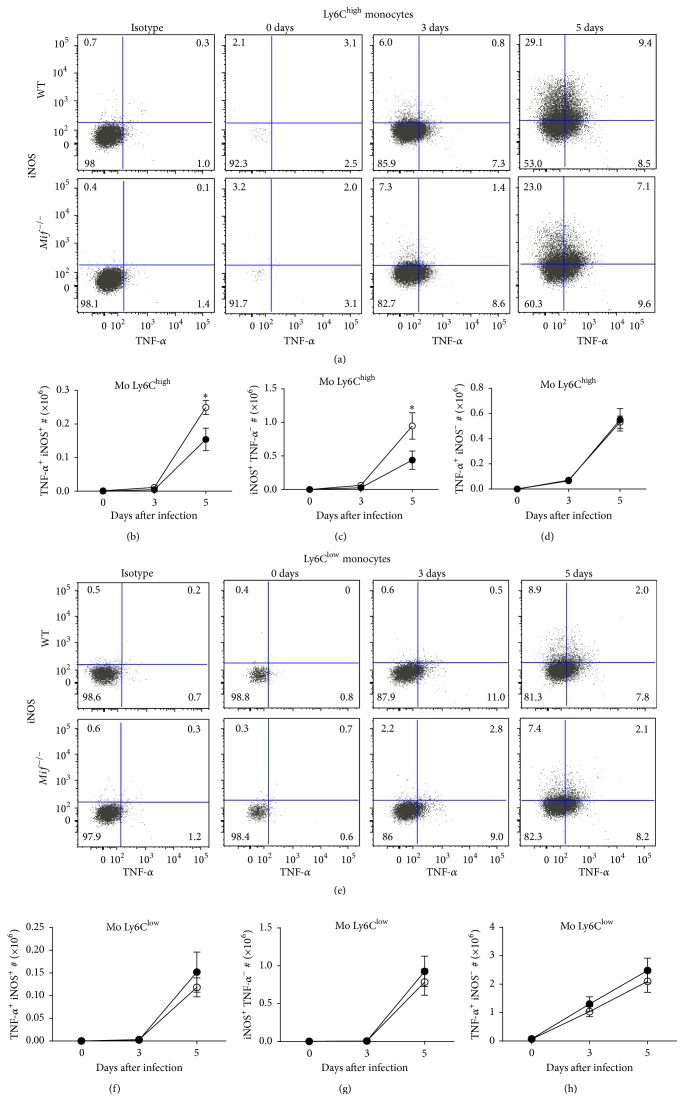
MIF promotes classical activation (M1) of inflammatory Ly6C^high^ monocytes. Monocytes (Ly6C^high^ and Ly6C^low^) from infected WT (○) and* Mif* ^−/−^ (●) mice were analyzed for iNOS and TNF-*α* production at 0, 3, and 5 days after infection. (a) Representative dot plots from iNOS/TNF-*α*-producing Ly6C^high^ monocytes. (b) Absolute numbers of iNOS^+^ TNF-*α* ^+^, (c) iNOS^+^ TNF-*α* ^−^, and (d) TNF-*α* ^+^iNOS^−^ cells from Ly6C^high^ monocytes. (e) Representative dot plots from iNOS/TNF-*α*-producing Ly6C^low^ monocytes. (f) Absolute numbers of iNOS^+^ TNF-*α* ^+^, (g) iNOS^+^ TNF-*α* ^−^, and (h) TNF-*α* ^+^iNOS^−^ cells from Ly6C^low^ monocytes, during the acute phase of infection with* T. gondii* (*n* = 3 animals at each time point, representative of 2 independent experiments). Data are presented as the mean ± SE. ^*∗*^ *P* < 0.05; ^*∗∗*^ *P* < 0.01.

We found that resident Ly6C^low^ monocytes and F4/80^high^ macrophages (M*ϕ*s) patrolled the peritoneal cavity in basal conditions (Figure S-2B, day 0). However, upon infection with* T. gondii*, newly recruited monocytes (Ly6C^high^ and Ly6C^low^) populated the peritoneal cavity at 3 and 5 days after infection, and few or no resident M*ϕ*s remained (Figure S-2B, 3 and 5 days after infection). No significant differences were observed in the absolute numbers of monocytes or resident M*ϕ*s between infected* Mif* ^−/−^ and WT mice (Figure S2C-E).  Figure 6 shows that* Mif* ^−/−^  Ly6C^high^ monocytes had reduced numbers of both TNF-*α* ^+^ iNOS^+^ (*P* = 0.0330; Figures 6(a) and 6(b)) and iNOS^+^ TNF-*α* ^−^ (*P* = 0.0473; Figures 6(a) and 6(c)) cells, when compared to WT Ly6C^high^ monocytes. No differences were observed in Ly6C^high^ TNF-*α* ^+^ iNOS^−^ monocytes between* Mif* ^−/−^ and WT infected mice (Figures 6(a) and 6(d)).

Interestingly, the Ly6C^low^ subset (Figure 6(e)) showed no differences between* Mif* ^−/−^ and WT infected mice in TNF-*α* ^+^ iNOS^+^ (Figure 6(f)), iNOS^+^ TNF-*α* ^−^ (Figure 6(g)), and TNF-*α* ^+^ iNOS^−^ monocytes. These data suggest that MIF promotes the activation of inflammatory Ly6C^high^ monocytes and possibly their differentiation into TipDCs during acute* T. gondii* infection.

### 3.6. MIF Induces Generation of TipDCs from Ly6C^high^ Monocytes during Acute* T. gondii* Infection

To address whether MIF influences differentiation of inflammatory Ly6C^high^ monocytes into TipDCs during the acute* T. gondii* infection, we determined the fate of adoptively transferred bone marrow Ly6C^high^ monocytes from* Mif* ^−/−^ or WT mice (donors) into* T. gondii*-infected (recipients)* Mif* ^−/−^ or WT mice (Figure S3).

**Figure 7:**
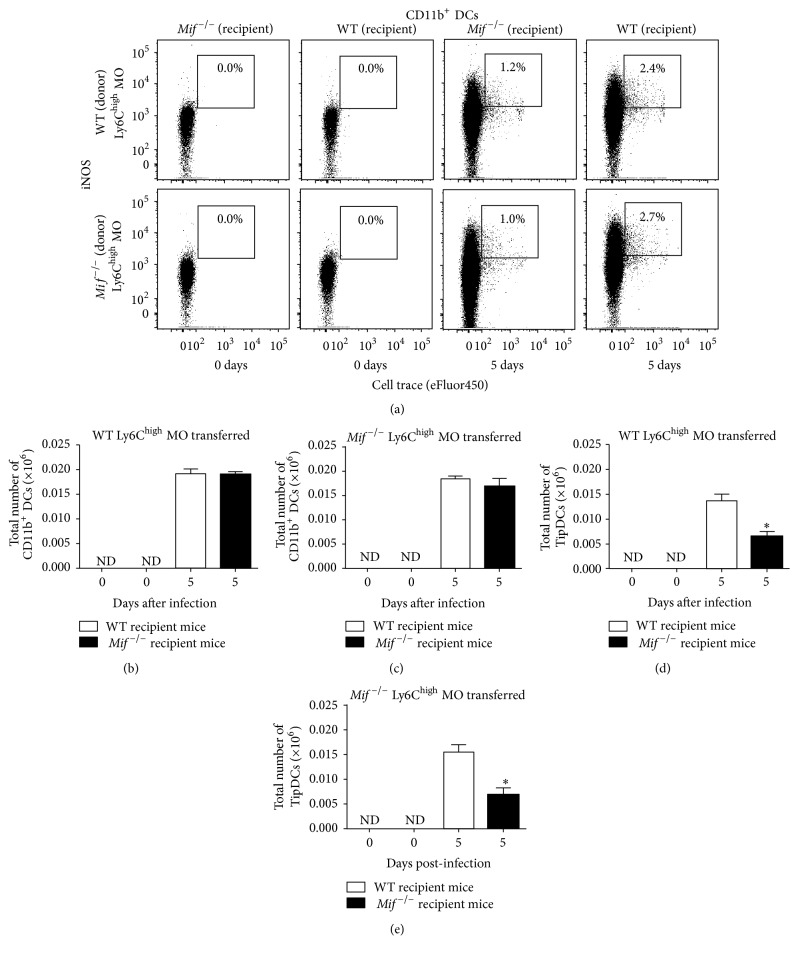
MIF promotes conversion of adoptively transferred Ly6C^high^ monocytes into TipDCs during acute* T. gondii* infection. Isolated WT or* Mif* ^−/−^  Ly6C^high^ monocytes were adoptively transferred into recipient* T. gondii*-infected WT or* Mif* ^−/−^mice and analyzed for CD11b^+^TipDC markers at 0 and 5 days after infection in the peritoneal cavity. (a) Representative dot plots from adoptively transferred WT or* Mif* ^−/−^  Ly6C^high^ monocytes differentiated into TipDCs. (b) Absolute numbers of adoptively transferred (cell trace-eFluor 450^+^ cells) WT Ly6C^high^ or (c)* Mif* ^−/−^  Ly6C^high^ monocytes differentiated into CD11b^+^ DCs (CD11c^+^ MHCII^+^ CD11b^+^). (d) Absolute numbers of adoptively transferred WT Ly6C^high^ or (e)* Mif* ^−/−^  Ly6C^high^ monocytes differentiated into TipDCs (CD11c^+^ MHCII^+^ CD11b^+^iNOS^+^). Data are representative of 2 independent experiments and are represented as the mean ± SE. ^*∗*^ *P* < 0.05; ^*∗∗*^ *P* < 0.01. ND: not detected.

We first analyzed the numbers of transferred Ly6C^high^ monocytes differentiated into CD11b^+^ DCs (CD11c^+^ MHCII^+^ CD11b^+^ cell trace^+^, Figure 7(a)) in the peritoneal cavity. No significant differences were observed in the total number of monocyte-derived CD11b^+^ DCs between WT and* Mif* ^−/−^ recipient mice after the transfer with WT or* Mif* ^−/−^  Ly6C^high^ monocytes (Figures 7(b) and 7(c), resp.). Moreover, we sought the TipDCs subset in the population of CD11b^+^ DCs and found that TipDCs were missing in both healthy WT and* Mif* ^−/−^ recipient mice (Figures 7(a), 7(d), and 7(e), day 0). However, after 5 days of* T. gondii* infection, WT and* Mif* ^−/−^ recipient mice showed a subset of Ly6C^high^ monocytes expressing markers for TipDCs (Figure 7(a), day 5). Interestingly, WT and* Mif* ^−/−^  Ly6C^high^ monocytes displayed similar ability to differentiate into TipDCs after being transferred to WT recipient mice (Figures 7(d) and 7(e), WT recipient mice). However, both WT and* Mif* ^−/−^  Ly6C^high^ monocytes were less able to differentiate into TipDCs when transferred into recipient* Mif* ^−/−^ mice compared to recipient WT mice (Figure 7(d), *P* = 0.0049; Figure 7(e), *P* = 0.0047). These results indicate that Ly6C^high^ monocytes differentiate into CD11b^+^ DCs in MIF-independent manner (Figures 7(b) and 7(c)), but endogenous MIF levels could be required to induce maturation of monocyte-derived CD11b^+^ DCs into TipDCs during acute toxoplasmosis.

On the other hand, Ly6C^high^ monocytes displayed high expression of MIF receptors CXCR4 (Figure S4: (A)-(B)) and CD74 (Figure S4: (C)-(D)), compared to Ly6C^low^ monocytes at 5 days after infection or resident M*ϕ*s (F4/80^high^) from uninfected mice, which could explain the selective role of MIF in inducing activation and differentiation of Ly6C^high^ monocytes into TipDCs. No differences were observed in CXCR2 expression (Figure S4: (E) and (F)).

### 3.7. MIF Promotes Interferon-*γ*-Producing NK Cells during Acute* T. gondii* Infection

**Figure 8:**
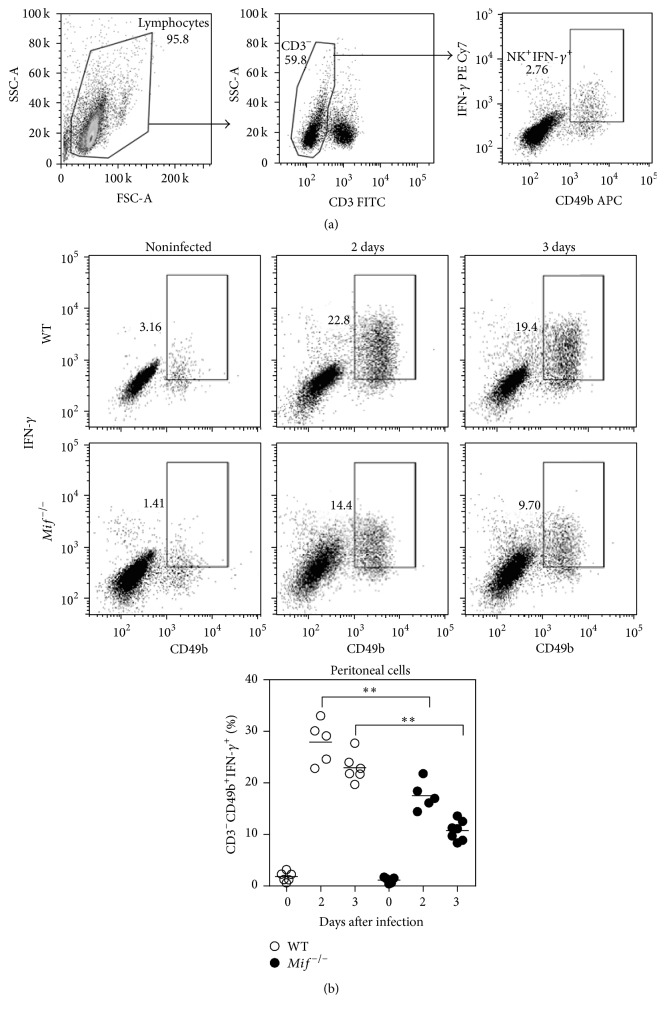
MIF promotes interferon-*γ*-producing NK cells during acute* T. gondii* infection. Peritoneal NK cells (CD3^−^ CD49b^+^) from WT (○) and* Mif* ^−/−^ (●) mice were analyzed for intracellular IFN-*γ* at 0, 2, and 3 days after* T. gondii* infection. (a) Gating strategy of peritoneal NK cells. (b) Representative FACs (upper panel) and percentages of IFN-*γ* ^+^ NK cells (lower panel) during the acute phase of infection with* T. gondii* (*n* = 5-6 animals at each time point, representative of 3 independent experiments). Data are presented as the mean ± SE. ^*∗∗*^ *P* < 0.01.

Early studies by Goldszmid et al. have demonstrated that natural killer (NK) cell-derived IFN-*γ* is responsible for driving the differentiation of mononuclear phagocytes into macrophages and IL-12 producing CD11b^+^ DCs during experimental toxoplasmosis [51]. In this context, the role of MIF in regulating NK cell-derived IFN-*γ* at the site of infection with* T. gondii* could be essential for the differentiation of Ly6C^high^ monocytes into TipDCs. Therefore, we analyzed the IFN-*γ* production in peritoneal NK cells from* T. gondii*-infected WT and* Mif* ^−/−^ mice; in Figure 8(a), we show the gating strategy used. We observed that either WT or* Mif* ^−/−^ NK cells (CD3^−^ CD49b^+^) displayed low percentages of IFN-*γ* ^+^ cells under steady state (Figure 8(b)). However, upon* T. gondii* infection, WT and* Mif* ^−/−^ NK cells (CD3^−^ CD49b^+^) drastically increased their IFN-*γ* production at 2 and 3 days after infection (Figure 8(b)). Interestingly,* Mif* ^−/−^ NK cells showed a reduced percentage of IFN-*γ* ^+^ cells, compared to WT NK cells (Figure 8(b)). This observation was in line with a higher number of* tachyzoites* observed in the peritoneum of* Mif* ^−/−^ compared with WT mice at days 5 and 7 after* T. gondii* infection (Figure S5).

The data suggest that MIF induces IFN-*γ*-producing NK cells during the acute infection with* T. gondii*. These findings could be related to a recent study in a murine model of skin inflammation, demonstrating a role for MIF in inducing the recruitment of IFN-*γ*-producing NKT cells [52]. Therefore, MIF could promote the migration and/or activation of NK cells during acute* T. gondii* infection, a crucial event in the differentiation of Ly6C^high^ monocytes into TipDCs.

### 3.8. rMIF Restores TipDCs and Resistance to* T. gondii* Infection in* Mif* ^−/−^ Mice

Because our results indicated that systemic MIF levels are critical for resistance and induction of TipDCs during acute toxoplasmosis, we confirmed these data by administration (via* i.p.*) of rMIF into* Mif* ^−/−^ mice (0.5 *μ*g per mouse) daily during the first 5 days after* T. gondii* infection.

**Figure 9:**
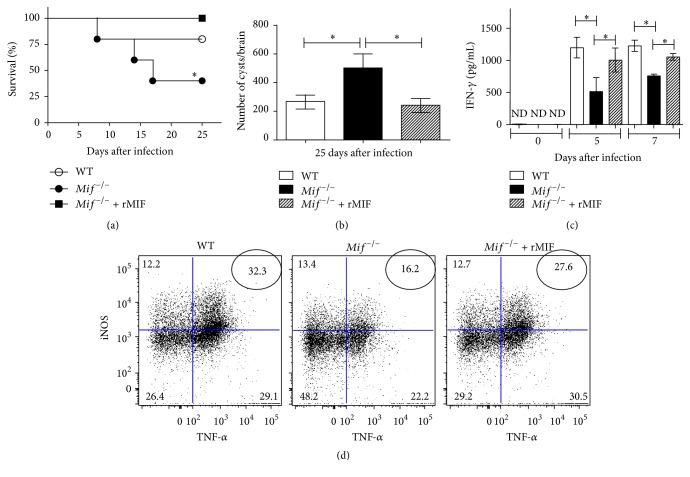
Administration of rMIF restores TipDCs and resistance to* T. gondii* infection in* Mif* ^−/−^mice.* T. gondii*-infected* Mif* ^−/−^ mice received 0.5 *μ*g of rMIF over the first 5 days of infection and were compared to infected WT and* Mif* ^−/−^ mice treated with PBS. (a) Survival rate of* T. gondii*-infected mice. (b) Number of cysts in brain from infected mice at 25 days after infection. (c) Serum IFN-*γ* levels from infected mice at 0, 5, and 7 days after infection. (d) Representative dot plots of TipDCs in the site of infection (*n* = 3 animals/group, representative of 2 independent experiments). Significant differences were calculated with the log rank test and Student's *t*-test. ND: not detected. Data are presented as the mean ± SE. ^*∗*^ *P* < 0.05.

The rMIF administration fully abolished the susceptibility of infected* Mif* ^−/−^ mice, which reached a survival rate of 100%, whereas untreated* Mif* ^−/−^ mice displayed a survival rate of 40% (*P* = 0.0114) (Figure 9(a)). The enhanced resistance induced by rMIF administration was associated with decreased numbers of cysts in the brain from* Mif* ^−/−^ mice (*P* = 0.0234; Figure 9(b)) after 25 days after infection. In addition, serum IFN-*γ* levels were significantly recovered in* Mif* ^−/−^ mice that received rMIF compared to* Mif* ^−/−^ that did not receive rMIF (*P* = 0.0107; Figure 9(c)). Strikingly, rMIF administration reconstituted the absolute numbers of TipDCs at 5 days after* T. gondii* infection (Figure 9(d)).

Altogether, these data demonstrate that induced MIF levels during acute toxoplasmosis have a crucial role in the resistance and the development of the protective cell-mediated immune response against* T. gondii*.

## 4. Discussion

We have previously demonstrated that MIF favors immunity to* T. gondii* by inducing inflammatory cytokines (IL-12 and IFN-*γ*) essential to control both acute and chronic infections with this parasite [24]. Moreover, MIF has a role in inducing maturation of CD11c^+^ cells [25], which indicates that MIF promotes cell-mediated immune responses against* T. gondii* infection. However, it is not known whether this inflammatory mediator induces maturation of specific DCs subsets required to control acute toxoplasmosis.

CD8*α* ^+^ and CD11b^+^ DCs have prominent roles as the major sources of the inflammatory cytokine IL-12, required for IFN-*γ*-mediated resistance to* T. gondii* [10–12, 31]. Therefore, in the present study, we sought to determine the role of MIF in the maturation of CD11b^+^ and CD8*α* ^+^ DCs during acute* T. gondii* infection. We found that MIF was one of the first cytokines increased in serum from infected WT mice, and in agreement with previous studies [24], the lack of this inflammatory mediator reduced the levels of other inflammatory cytokines, such as IL-12, IFN-*γ*, and TNF-*α*, with all of them involved in immunity against* T. gondii* [32, 53–55].

Interestingly, CD11b^+^ but not CD8*α* ^+^ DCs from infected* Mif* ^−/−^ mice showed impaired maturation characterized by deficient IL-12p70 production and low expression of the costimulatory molecule CD86. Therefore, as CD11b^+^ DCs have a crucial role in the control of acute toxoplasmosis [10], an important mechanism by which MIF promotes resistance to* T. gondii* may be through inducing maturation of CD11b^+^ DCs. Additionally, CD11b^+^ DCs displayed high expression of MIF receptors CXCR4, CD74, and CXCR2 [56] compared to CD8*α* ^+^ DCs, suggesting that CD11b^+^ DCs are more sensitive to paracrine/autocrine MIF stimulus than CD8*α* ^+^ DCs during acute toxoplasmosis.

Parasitic or microbial infections with* Trypanosoma brucei* [38, 39] or* Listeria monocytogenes* [36], respectively, induce potent inflammatory immune responses that generate iNOS/TNF-*α*-producing CD11b^+^ DCs (TipDCs), which derive from inflammatory monocytes recruited in the site of infection. Similarly,* i.p. T. gondii* infection induces monocyte-derived IL-12 producing CD11b^+^ DCs, which accumulate in the peritoneal cavity and have a crucial role in the protective immune response to the parasite [10]. Nevertheless, the production of iNOS and TNF-*α*, essential factors in the control of* T. gondii* infection [4, 32–35], has not been addressed on this DC subset [32, 49, 57, 58]. Here, we showed that, during acute* i.p.* infection with* T. gondii*, a population of TipDCs gradually increased in the peritoneal cavity from infected WT and* Mif* ^−/−^ mice. Interestingly, infected* Mif* ^−/−^ mice displayed reduced numbers of TipDCs (5 days after infection) compared to infected WT mice, indicating a novel role for MIF in inducing TipDCs during experimental toxoplasmosis. However, it is important to note that although the term TipDCs has been widely accepted to designate iNOS/TNF-*α*-producing DCs, previous reports have argued that it does not necessarily refer to a distinct DC subset, but rather to common DCs responding to their environment with iNOS and TNF-*α* production [36, 37]. Therefore, as comparable numbers of CD11b^+^ DCs were detected in both infected* Mif* ^−/−^ and WT mice, but reduced numbers of TipDCs were observed in the absence of MIF, these data suggest that MIF induces maturation of CD11b^+^ DCs into TipDCs, rather than recruitment of TipDCs.

Furthermore, a small population of CD8*α* ^+^ DCs was detected during acute* T. gondii* infection (Figure 4(b)), corroborating a previous report [10]. CD8*α* ^+^ DCs displayed MIF-independent maturation, characterized by increased TNF-*α* but not iNOS production, which also indicates that CD11b^+^ but not CD8*α* ^+^ DCs are able to become TipDCs during acute toxoplasmosis.

Previous studies have demonstrated that NK cell-derived IFN-*γ* is required for local differentiation of inflammatory monocytes into both macrophages and mature IL-12 producing DCs [10]. In contrast, maturation of CD8*α* ^+^ DCs does not require priming by IFN-*γ* [31, 59] but is critically dependent on TLR stimuli. Interestingly, the role of MIF inducing the recruitment of IFN-*γ*-producing NKT cells in a murine model of psoriasis has been recently demonstrated [52]. Therefore, we analyzed IFN-*γ*-producing NK cells from* T. gondii*-infected WT and* Mif* ^−/−^ mice. We found that* T. gondii*-infected* Mif* ^−/−^ mice displayed a lower percentage of IFN-*γ* ^+^ NK cells compared to infected WT mice, which could alter the activation and local differentiation of inflammatory monocytes into TipDCs.

Inflammatory monocytes are known as the main precursors of TipDCs during parasitic and microbial infections [36, 39]. A variety of studies indicate that Ly6C^high^ monocytes are required to control cerebral toxoplasmosis [50], and depletion of inflammatory monocytes (Ly6C^+^) but not neutrophils (Ly6G^+^) increases the susceptibility of C57BL/6 mice to oral* T. gondii* infection [46–49]. Therefore, we analyzed the activation state and ability of inflammatory monocytes to differentiate into TipDCs during acute toxoplasmosis. We observed that, upon* i.p.* infection with* T. gondii*, newly recruited monocytes (Ly6C^high^ and Ly6C^low^) populated the peritoneal cavity of infected mice in a MIF-independent manner. However, the absence of MIF reduced the activation of Ly6C^high^ monocytes as indicated by significant lower iNOS/TNF-*α* production (well-established markers for M1 polarization). In contrast, this was not observed on Ly6C^low^ monocytes, suggesting a role for MIF in promoting the activation of Ly6C^high^ monocytes into a M1 profile. Furthermore, adoptive transfer of bone marrow Ly6C^high^ monocytes revealed that* Mif* ^−/−^ monocytes were as capable as WT monocytes of converting into TipDCs when transferred to infected WT mice. However, this ability was reduced in both, WT and* Mif* ^−/−^ monocytes, when transferred into infected* Mif* ^−/−^ mice, demonstrating that the presence of MIF in Ly6C^high^ monocytes has a limited role in the conversion of this population into TipDCs, compared to systemic MIF levels released/produced by other cell types during acute* T. gondii* infection.

Finally, administration of rMIF to infected* Mif* ^−/−^ mice over the first 5 days of infection with* T. gondii* fully restored the resistance of* Mif* ^−/−^ mice and reconstituted the absolute numbers of TipDCs as well as serum IFN-*γ* levels. Enhanced resistance by rMIF administration was associated with reduced parasite loads in brain, which indicated a better control of* T. gondii* infection.

## 5. Conclusions

Our data demonstrate a novel role for MIF promoting the resistance to* T. gondii*-infection by inducing classical activation of inflammatory monocytes Ly6C^high^ and their conversion into TipDCs. In addition, MIF induced IFN-*γ*-producing NK cells during the acute phase of infection with* T. gondii*, required for differentiation of inflammatory monocytes into TipDCs. Further experiments are required to detail the mechanism by which MIF regulates IFN-*γ* production in NK cells.

The administration of rMIF into* Mif* ^−/−^ mice fully restored the resistance to acute toxoplasmosis, which highlights the crucial role of this inflammatory mediator in the development of the protective cell-mediated immune response to* T. gondii* and indicates a potential use of rMIF as a therapeutic tool on immune-compromised hosts to control toxoplasmosis and probably other protozoan infectious diseases.

## Supplementary Material

Figure S1. MIF induces maturation of CD11b+ but not CD8α+ DCs in spleen from Mif-/- mice infected with T. gondii. WT and Mif-/- mice were intraperitoneally infected with 100 cysts of ME49 T. gondii strain, sacrificed on days 0, 1, 2, 3, 5 and 7 after infection, and analyzed for extracellular CD86 and intracellular IL-12 in dendritic cell subsets by flow cytometry. (A) Absolute numbers of CD11b+ (B) and CD8α+ DCs. (C) Absolute numbers of IL-12p70+ CD11b+ DCs. (D) Representative flow cytometry dot plots gated on MHCII+ CD11c+ CD11b+ DCs. (E) Absolute numbers of IL-12p70+ CD8α+. (F) Representative flow cytometry dot plots gated on MHCII+ CD11c+ CD8α+ DCs. (G) Absolute numbers of CD86+ CD11b+ and (H) CD86+ CD8α+ DCs in spleen from WT (white circles) and Mif-/- (black circles) mice, throughout the course of infection (n=3 animals at each time point, representative of 3 independent experiments). Data are represented as mean +/- SE. ∗ P< 0.05.
Figure S2. Acute experimental toxoplasmosis induces recruitment of Ly6Chigh and Ly6Clow monocytes. Macrophage (Mϕs) and monocyte subsets were characterized in PECs from infected WT and Mif-/- mice at 0, 3 and 5 days after T. gondii infection. (A) Gating strategy of macrophages (Ly6G- CD11b+ F480high) and monocytes (Ly6G- CD11b+ Ly6Chigh or Ly6Clow). (B) Representative dotplots of monocytes Ly6Chigh, Ly6Clow and macrophages F480high, during the acute T. gondii infection. (C) Absolut numbers of monocytes Ly6Chigh, (D) Ly6Clow and (E) Mϕs F480high throughout the course of infection (n=3 animals at each time point, representative of 2 independent experiments). Data are presented as mean +/- SE.
Figure S3. Monocyte adoptive transfer. (A) WT or Mif-/- bone marrow monocytes CD49b- CD90- Ly6G- CD11b+ Ly6C+ were isolated by negative selection, (B) separated in Ly6Chigh and Ly6Clow monocytes by cell sorting. (C) Ly6Chigh monocytes were labeled with efluor 450 dye, (D) and transferred via i.v. into WT and Mif-/- mice infected with T. gondii-ME49 strain. (E) Five days after infection PECs were harvested and analyzed by flow cytometry.
Figure S4. Expression of MIF receptors CXCR2, CXCR4 and CD74 in monocytes (Ly6Chigh and Ly6Clow) and macrophages (F480high). Macrophages (Mϕs F4/80high) and monocytes (Mo Ly6Chigh and Ly6Clow) from infected WT and Mif-/- mice were analyzed for CXCR2, CXCR4 and CD74 expression. (A) Bars of CXCR4, (C) CD74 and (E) CXCR2 MFI in macrophages from uninfected mice and monocytes Ly6Chigh and Ly6Clow at 5 days after T. gondii infection. Representative histograms of (B) CXCR4 (D) CD74 and (F) CXCR2 expression on Mϕs and Mos Ly6Chigh and Ly6Clow (n=3 animals at each time point, representative of 2 independent experiments). Data are shown as mean +/- SE. ∗ P< 0.05.
Figure S5. Local peritoneal parasite load was increased in Mif-/- mice. To assess acute disease progression, the peritoneal fluids from Mif-/- and WT mice were recovered under steril condition at days 5 and 7 after intraperitoneal infection with 100 cyst of T. gondii. Tachyzoites were counted with a Neubauer hemocytometer under Olympus BX51 microscope (X100; Olympus American, Melville, NY) equipped with a digital video camera. Data are shown as mean (x104) +/- SE (n=4 animals). ∗ P< 0.05 compared to WT infected mice at each time point.
