# Supplementary material for: MIF Promotes Classical Activation and Conversion of Inflammatory Ly6Chigh Monocytes into TipDCs during Murine Toxoplasmosis
Source: Mediators Inflamm. 2016 Feb 29;2016:9101762. doi: 10.1155/2016/9101762 (PMC4789477; doi:10.1155/2016/9101762)
Supplement: Supplementary file 1 — Figure S1. MIF induces maturation of CD11b+ but not CD8α+ DCs in spleen from Mif-/- mice infected with T. gondii. WT and Mif-/- mice were intraperitoneally infected with 100 cysts of ME49 T. gondii strain, sacrificed on days 0, 1, 2, 3, 5 and 7 after infection, and analyzed for extracellular CD86 and intracellular IL-12 in dendritic cell subsets by flow cytometry. (A) Absolute numbers of CD11b+ (B) and CD8α+ DCs. (C) Absolute numbers of IL-12p70+ CD11b+ DCs. (D) Representative flow cytometry dot plots gated on MHCII+ CD11c+ CD11b+ DCs. (E) Absolute numbers of IL-12p70+ CD8α+. (F) Representative flow cytometry dot plots gated on MHCII+ CD11c+ CD8α+ DCs. (G) Absolute numbers of CD86+ CD11b+ and (H) CD86+ CD8α+ DCs in spleen from WT (white circles) and Mif-/- (black circles) mice, throughout the course of infection (n=3 animals at each time point, representative of 3 independent experiments). Data are represented as mean +/- SE. ∗ P< 0.05. Figure S2. Acute experimental toxoplasmosis induces recruitment of Ly6Chigh and Ly6Clow monocytes. Macrophage (Mϕs) and monocyte subsets were characterized in PECs from infected WT and Mif-/- mice at 0, 3 and 5 days after T. gondii infection. (A) Gating strategy of macrophages (Ly6G- CD11b+ F480high) and monocytes (Ly6G- CD11b+ Ly6Chigh or Ly6Clow). (B) Representative dotplots of monocytes Ly6Chigh, Ly6Clow and macrophages F480high, during the acute T. gondii infection. (C) Absolut numbers of monocytes Ly6Chigh, (D) Ly6Clow and (E) Mϕs F480high throughout the course of infection (n=3 animals at each time point, representative of 2 independent experiments). Data are presented as mean +/- SE. Figure S3. Monocyte adoptive transfer. (A) WT or Mif-/- bone marrow monocytes CD49b- CD90- Ly6G- CD11b+ Ly6C+ were isolated by negative selection, (B) separated in Ly6Chigh and Ly6Clow monocytes by cell sorting. (C) Ly6Chigh monocytes were labeled with efluor 450 dye, (D) and transferred via i.v. into WT and Mif-/- mice infected with T. gondii-ME4 [file 9101762.f1.pptx]

## Slide 1
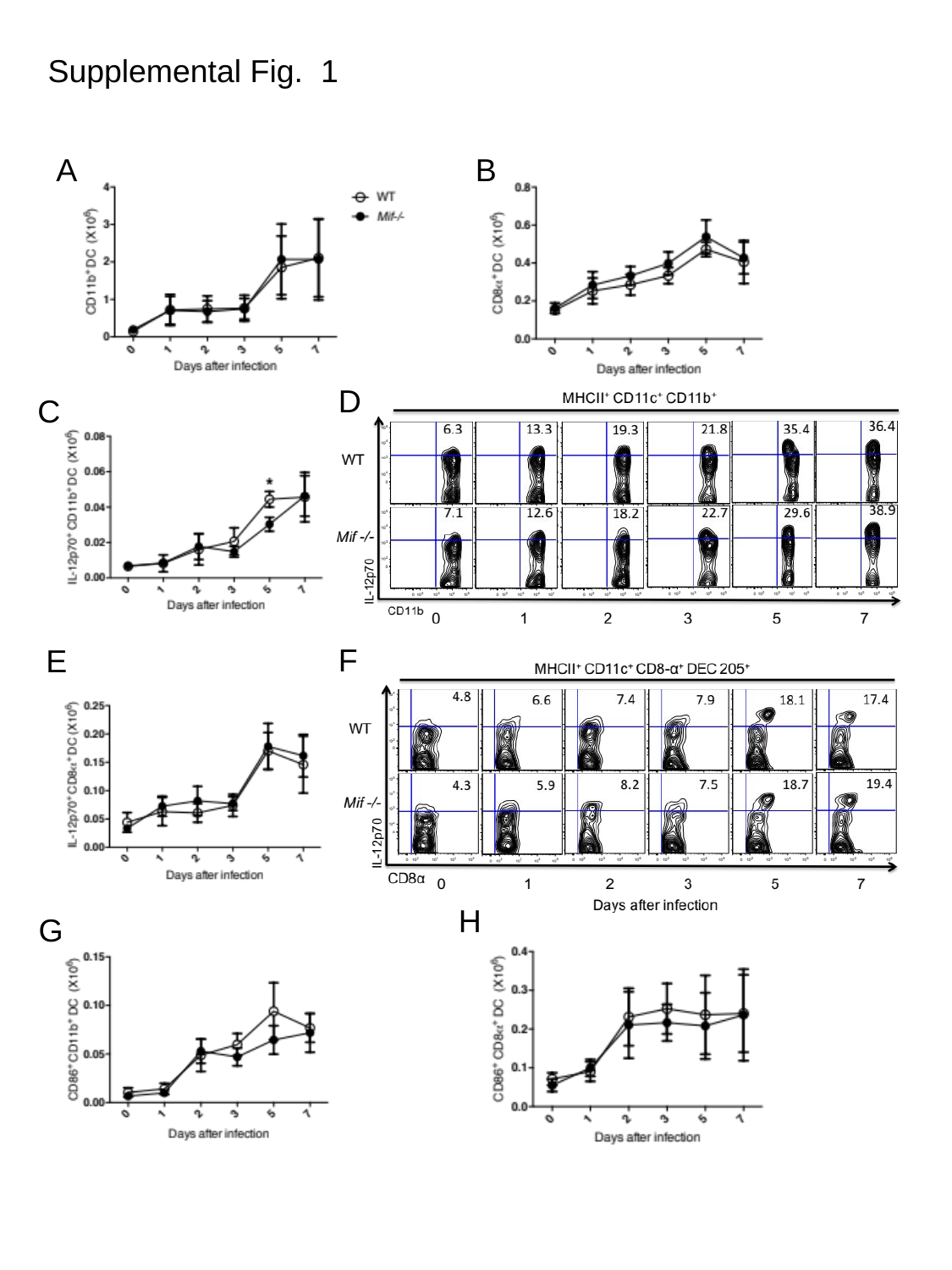

Supplemental Fig. 1
A
B
D
C
F
E
H
G

## Slide 2
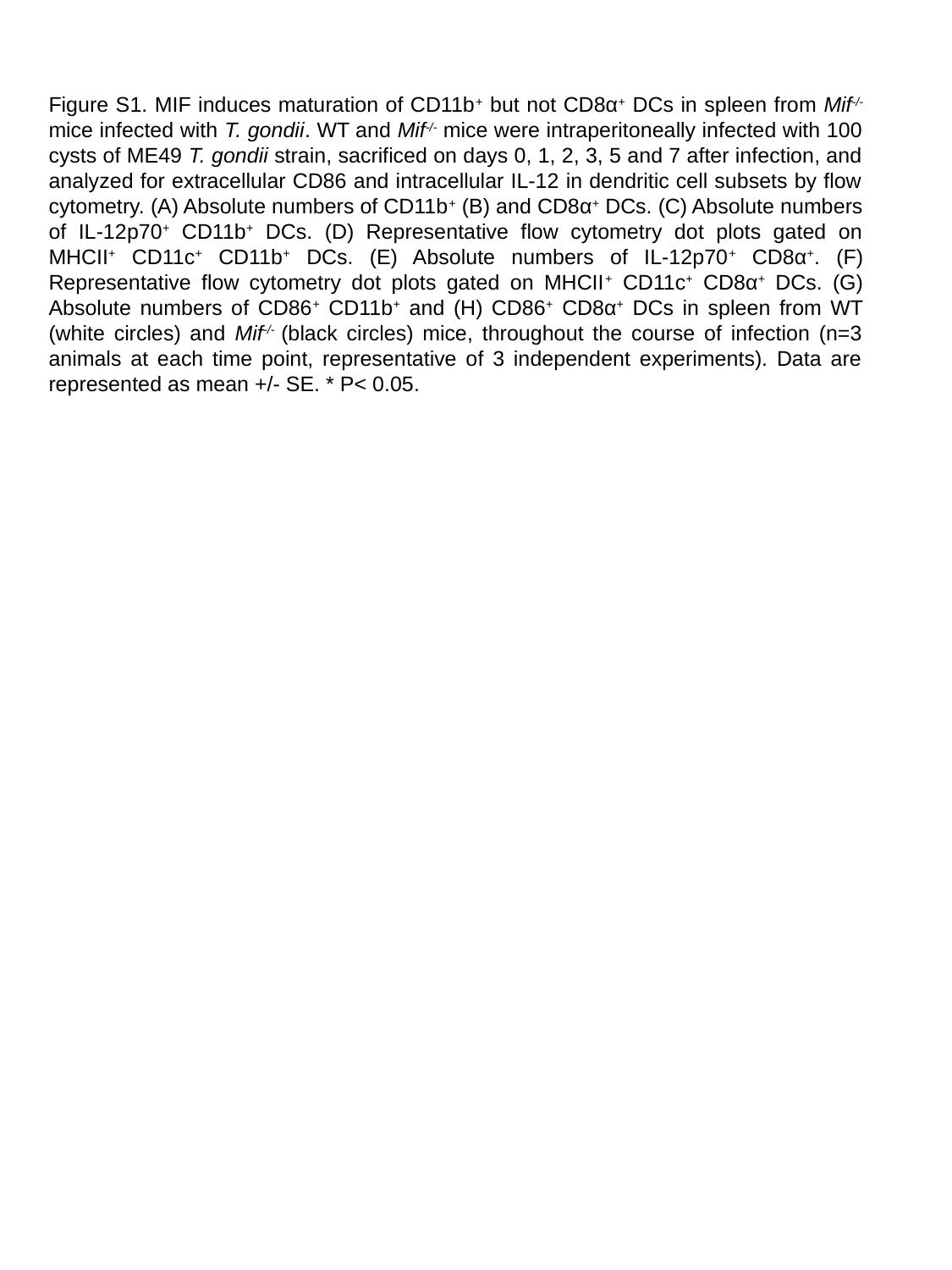

Figure S1. MIF induces maturation of CD11b+ but not CD8α+ DCs in spleen from Mif-/- mice infected with T. gondii. WT and Mif-/- mice were intraperitoneally infected with 100 cysts of ME49 T. gondii strain, sacrificed on days 0, 1, 2, 3, 5 and 7 after infection, and analyzed for extracellular CD86 and intracellular IL-12 in dendritic cell subsets by flow cytometry. (A) Absolute numbers of CD11b+ (B) and CD8α+ DCs. (C) Absolute numbers of IL-12p70+ CD11b+ DCs. (D) Representative flow cytometry dot plots gated on MHCII+ CD11c+ CD11b+ DCs. (E) Absolute numbers of IL-12p70+ CD8α+. (F) Representative flow cytometry dot plots gated on MHCII+ CD11c+ CD8α+ DCs. (G) Absolute numbers of CD86+ CD11b+ and (H) CD86+ CD8α+ DCs in spleen from WT (white circles) and Mif-/- (black circles) mice, throughout the course of infection (n=3 animals at each time point, representative of 3 independent experiments). Data are represented as mean +/- SE. * P< 0.05.

## Slide 3
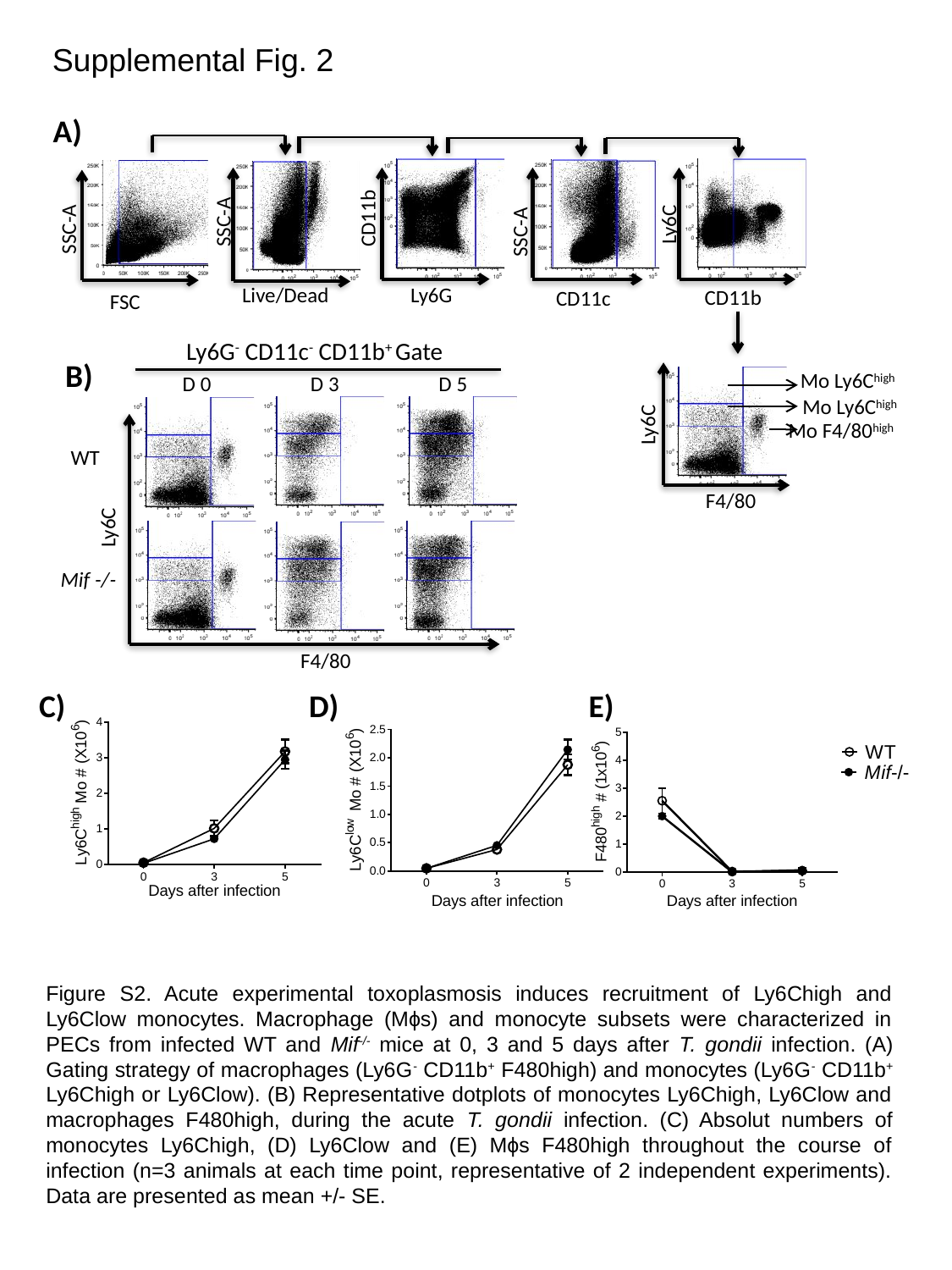

Supplemental Fig. 2
A)
CD11b
Ly6C
SSC-A
SSC-A
SSC-A
Live/Dead
Ly6G
CD11b
CD11c
FSC
Ly6G- CD11c- CD11b+ Gate
B)
Mo Ly6Chigh
Ly6C
F4/80
D 0
D 3
D 5
Mo Ly6Chigh
Mo F4/80high
WT
Ly6C
Mif -/-
F4/80
C)
D)
E)
Figure S2. Acute experimental toxoplasmosis induces recruitment of Ly6Chigh and Ly6Clow monocytes. Macrophage (Mϕs) and monocyte subsets were characterized in PECs from infected WT and Mif-/- mice at 0, 3 and 5 days after T. gondii infection. (A) Gating strategy of macrophages (Ly6G- CD11b+ F480high) and monocytes (Ly6G- CD11b+ Ly6Chigh or Ly6Clow). (B) Representative dotplots of monocytes Ly6Chigh, Ly6Clow and macrophages F480high, during the acute T. gondii infection. (C) Absolut numbers of monocytes Ly6Chigh, (D) Ly6Clow and (E) Mϕs F480high throughout the course of infection (n=3 animals at each time point, representative of 2 independent experiments). Data are presented as mean +/- SE.

## Slide 4
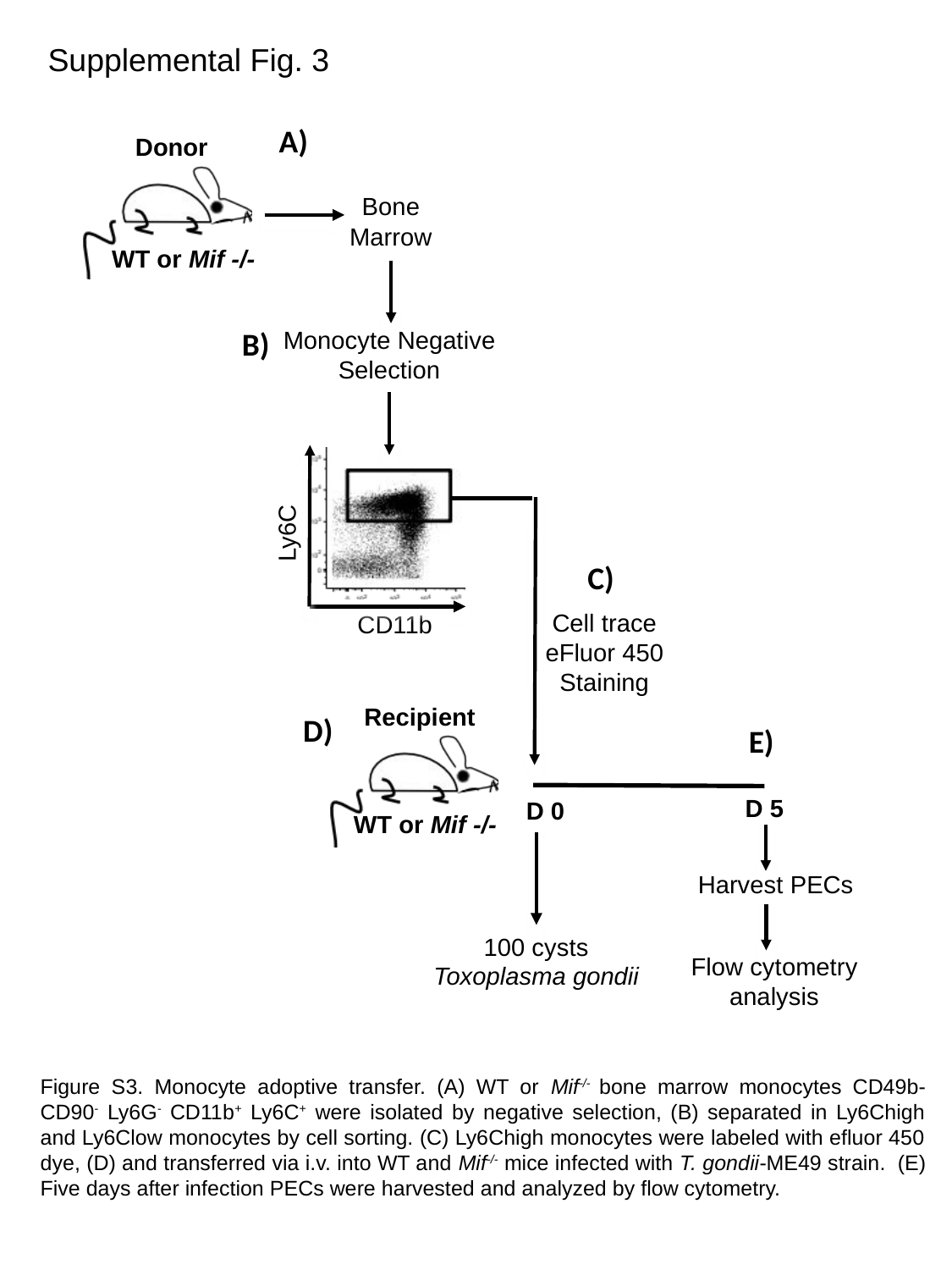

Supplemental Fig. 3
A)
Donor
Bone
Marrow
WT or Mif -/-
B)
Monocyte Negative
Selection
Ly6C
C)
Cell trace
eFluor 450
Staining
CD11b
Recipient
D)
E)
D 5
D 0
WT or Mif -/-
Harvest PECs
100 cysts
Toxoplasma gondii
Flow cytometry
analysis
Figure S3. Monocyte adoptive transfer. (A) WT or Mif-/- bone marrow monocytes CD49b- CD90- Ly6G- CD11b+ Ly6C+ were isolated by negative selection, (B) separated in Ly6Chigh and Ly6Clow monocytes by cell sorting. (C) Ly6Chigh monocytes were labeled with efluor 450 dye, (D) and transferred via i.v. into WT and Mif-/- mice infected with T. gondii-ME49 strain. (E) Five days after infection PECs were harvested and analyzed by flow cytometry.

## Slide 5
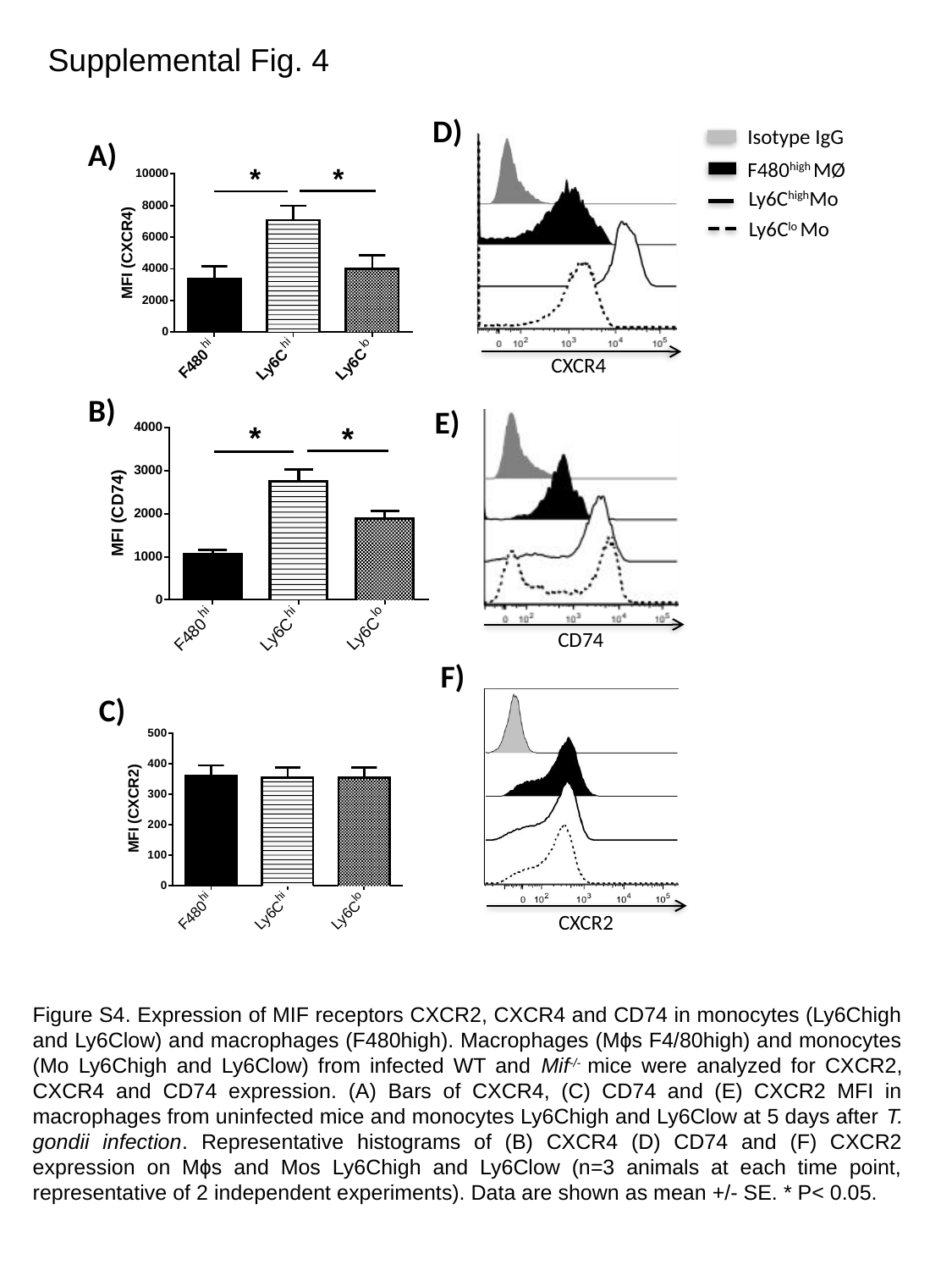

Supplemental Fig. 4
D)
Isotype IgG
F480high MØ
Ly6ChighMo
Ly6Clo Mo
A)
CXCR4
B)
E)
CD74
F)
C)
CXCR2
Figure S4. Expression of MIF receptors CXCR2, CXCR4 and CD74 in monocytes (Ly6Chigh and Ly6Clow) and macrophages (F480high). Macrophages (Mϕs F4/80high) and monocytes (Mo Ly6Chigh and Ly6Clow) from infected WT and Mif-/- mice were analyzed for CXCR2, CXCR4 and CD74 expression. (A) Bars of CXCR4, (C) CD74 and (E) CXCR2 MFI in macrophages from uninfected mice and monocytes Ly6Chigh and Ly6Clow at 5 days after T. gondii infection. Representative histograms of (B) CXCR4 (D) CD74 and (F) CXCR2 expression on Mϕs and Mos Ly6Chigh and Ly6Clow (n=3 animals at each time point, representative of 2 independent experiments). Data are shown as mean +/- SE. * P< 0.05.

## Slide 6
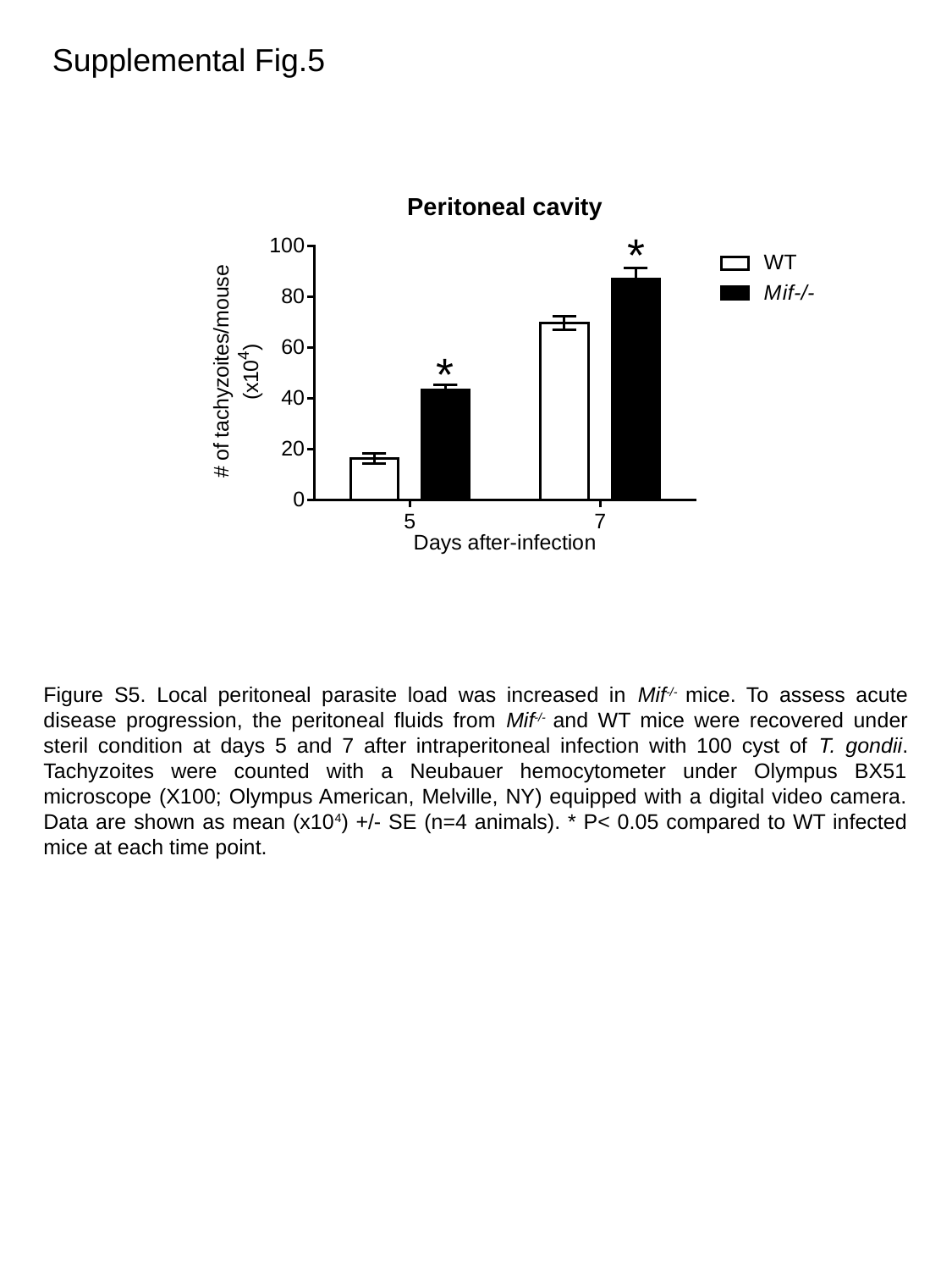

Supplemental Fig.5
Figure S5. Local peritoneal parasite load was increased in Mif-/- mice. To assess acute disease progression, the peritoneal fluids from Mif-/- and WT mice were recovered under steril condition at days 5 and 7 after intraperitoneal infection with 100 cyst of T. gondii. Tachyzoites were counted with a Neubauer hemocytometer under Olympus BX51 microscope (X100; Olympus American, Melville, NY) equipped with a digital video camera. Data are shown as mean (x104) +/- SE (n=4 animals). * P< 0.05 compared to WT infected mice at each time point.
